# Midterm Results of Precuffed Grafts in Lower Limb Bypass Surgeries

**DOI:** 10.7759/cureus.82142

**Published:** 2025-04-12

**Authors:** Ibraheem Obaidat, Nyagan Kalam-Sakit, Ahmed Elbadawy, Sarah Michael, Walid Alnatsheh, Mohammed Aymen Alsheikh, Ferdinand Serracino-Inglott, Kamran Khan

**Affiliations:** 1 Vascular Surgery, Manchester Royal Infirmary, Manchester, GBR; 2 General Surgery, Jordan University Hospital, Amman, JOR; 3 Vascular Surgery, Manchester University Hospitals, Manchester, GBR; 4 General and Colorectal Surgery, Manchester University Hospitals, Manchester, GBR

**Keywords:** amputation-free survival, chronic limb ischemia, distaflo, limb ischemia, lower limb bypasses, precuffed vascular grafts, vascular surgery

## Abstract

Background

Prosthetic grafts have been used for lower limb bypass surgery when the long saphenous vein option is not available; however, they showed suboptimal outcomes, especially when used below the knee. The use of precuffed grafts has emerged as a potential solution to address some of the limitations associated with traditional graft choices.

Materials and methods

All patients who underwent bypass surgery using precuffed grafts in Manchester Royal Infirmary between March 2020 and August 2023 were included in the retrospective analysis. The primary outcome was amputation-free survival over one month, 12 months, and 24 months. Secondary outcomes were mortality rate, patency rate, and postoperative complications.

Results

A total of 87 patients were included in this study; 47.1% of patients achieved the primary outcome endpoint by the end of 24 months. Smoking (hazard ratio {HR} 4.103, CI 1.417-11.881, p=0.009), female sex (HR 0.304, CI 0.119-0.774, p=0.013), and presence of tissue loss (HR 0.217, CI 0.086-0.55, p=0.001) were independent statistically significant predictors of poor primary outcome of precuffed grafts.

The mortality rate was 20.7% at 24 months, with cardiac disease as predictive of a higher mortality rate; the patency rate was 41.4% at 24 months. The following were negative predictors for graft patency: smoking, female sex, and presence of tissue loss.

Conclusion

While the long saphenous vein remains the preferred choice for bypass, our study demonstrates that precuffed grafts are a valid alternative when the long saphenous vein is unavailable. These grafts show an acceptable amputation-free survival rate over a two-year follow-up period.

## Introduction

Lower limb bypass surgery plays a vital role in limb salvage for patients experiencing lower limb ischemia, which can arise from a variety of causes, including progressive atherosclerosis, diabetes, embolism, trauma, and aneurysms. The ideal bypass conduit should resemble the native vessel, be accessible, durable, easy to handle, affordable, and resistant to infection and thrombosis [1]. While no ideal conduit exists, the saphenous vein is often used due to its favourable characteristics. However, it may be unavailable (previously harvested) or unsuitable (small or varicosed).

Synthetic grafts offer acceptable outcomes, especially above the knee, but are less effective than saphenous vein grafts below the knee [2,3]. Despite advances in endovascular management, we still encounter cases where this option is limited, such as in cases of extensive disease with unfavorable outcomes, previous failed attempts, or complications related to endovascular treatment that require an alternative bailout procedure.

Precuffed grafts are a promising alternative. These specialized grafts are made of expanded polytetrafluoroethylene (ePTFE) and feature a cuff at one end, designed to facilitate the distal anastomosis and reduce endothelial damage and intimal hyperplasia, potentially improving long-term patency. The size of the cuff can vary depending on the target vessel for the distal anastomosis [4,5]. This study evaluates the midterm result of precuffed grafts by assessing amputation-free survival at 30 days, 12 months, and 24 months.

## Materials and methods

Patient selection

A retrospective review was conducted, including all patients who underwent lower limb bypass surgery using a precuffed graft between March 2020 and August 2023 at Manchester Royal Infirmary. Data were retrieved using the hospital electronic database and local registry, which contain patients' hospital numbers, date of the procedure, name of the procedure, and type of graft used.

Institutional review board approval and patient consent were not required since this was a retrospective study of prospectively collected information on an electronic database regarding outcomes following procedures and reviewing patient case notes. There is no patient identifiable data presented, and there is no way of identifying individual patients and therefore, patient consent was not required. This work has been reported in line with the Strengthening the Reporting of Observational Studies in Epidemiology (STROBE) criteria [6].

Patient characteristics, including age, sex, indication for surgery, type of surgery (emergency vs. elective), cardiac disease, diabetes, arterial hypertension, smoking status, bypass target vessel, previous bypass on the same limb, and clinical frailty score, were retrieved from the patients' electronic medical charts.

All patients who underwent lower limb bypass using precuffed grafts were included in the study. The study encompassed patients with various indications for lower limb bypass, including acute limb ischemia, chronic limb-threatening ischemia, claudication, and popliteal aneurysm. Patients were excluded if they underwent bypass with standard prosthetic grafts or upper limb bypass with precuffed grafts.

Outcomes

The primary outcome was defined as amputation-free survival at 30 days, 12 months, and 24 months. Amputation-free survival was defined as the length of time a patient survives after the index procedure without undergoing above-ankle amputation of the index limb. Secondary outcomes included mortality rate, patency rate, and postoperative complications (infection, bleeding, and pseudoaneurysm formation); we chose those postoperative complications as they were the most commonly reported in our cohort of patients.

Surgical procedure

All patients had at least one imaging modality to determine the level of occlusion and the target vessel. Also, all cases were managed at a tertiary vascular centre with 24/7 access to a hybrid theatre. Both elective and emergency cases had a vein map ultrasound assessment of ipsilateral and contralateral limb before considering the use of a prosthetic graft.

All elective patients were on high dose of statin and antiplatelet. A prophylactic dose of intravenous antibiotic was administered before the surgical incision, following local prophylactic antibiotic guidelines. Standard femoral artery and target artery exposure were performed, along with the creation of an anatomical tunnel. Intravenous unfractionated heparin was given based on the patient's weight, and anastomoses were carried out in accordance with the manufacturer’s instructions for use [7].

The instructions for use clearly recommend not modifying the size of the cuff. Instead, different cuff configurations should be used, as the grafts are available with standard, small, and mini cuffs [7]. The choice of the target artery is based on selecting the optimal vessel to ensure in-line blood flow to the foot. The arteriotomy size must correspond to the cuff size, and the suture materials used are standard non-absorbable monofilament sutures on a non-cutting needle. Postoperative management included establishing the best medical treatment in all patients, which consists of a high dose of statin, antiplatelet, glycemic control, and smoking cessation recommendations.

Follow-up

Patients’ outcomes were checked on 30 days, 12 months, and 24 months from the index procedure using the hospital electronic database and general practitioner records. Data collected included the occurrence of above-ankle amputations, date of death (if applicable), postoperative complications, and graft patency status (determined by imaging when available).

Data analysis

Descriptive statistics were used with continuous variables expressed as mean±standard deviation (SD) and/or median and interquartile range (IQR), and categorical variables as frequency and percentage. Categorical variables were compared using the chi-square test or Fisher's exact test, while continuous variables were compared using Student's t-test or Analysis of Variance (ANOVA). Multivariable Cox proportional hazard regression, including patient and lesion characteristics, was performed, with a p-value of 0.05 indicating a statistically significant relationship.

## Results

A total of 87 patients underwent lower limb bypass using precuffed graft at Manchester Royal Infirmary Hospital between March 2020 and August 2024. The indications for surgery were chronic limb-threatening ischemia (85%, n=74) (i.e., gangrene, ischemic rest pain, diabetic foot ulcer, or any form of lower limb ulceration present for more than two weeks), acute limb ischemia (12%, n=10), disabling claudication (2%, n=2), and popliteal aneurysm (1%, n=1) (Table 1) [8].

**Table 1:** Patients' baseline characteristics. CFS: clinical frailty score; CLTI: chronic limb-threatening ischemia; ALI: acute limb ischemia; IC: intermittent claudication; N: total number of patients Data are presented as n (%) and mean and range for continuous variables.

Variables (N=87)	n (%)
Sex	
Male	56 (64.4%)
Female	31 (35.6%)
Age mean (range in years)	69.1 (44-88)
Diabetic	35 (40.2%)
Hypertension	53 (60.9%)
Cardiac disease	40 (46.0%)
Indication	
ALI	10 (11.5%)
CLTI	74 (85.1%)
Disabling IC	2 (2.3%)
Popliteal aneurysm	1 (1.1%)
Smoker	43 (49.4%)
CFS	
1	1 (1.1%)
3	5 (5.7%)
4	27 (31%)
5	15 (17.2%)
6	26 (29.9%)
7	2 (2.3%)
Missing	11 (12.6%)
Redo surgery	23 (26.4%)
Emergency	42 (48.3%)
Tissue loss	54 (62.1%)
Target artery	
Popliteal	57 (65.5%)
Tibial	30 (34.5%)

The mean age of patients was 69 years, most of them were males (64%, n=56). Our patients’ comorbidities resemble the known risk factors of peripheral vascular diseases including diabetes (40%, n=35), hypertension (61%, n=53), and ischemic heart disease (46%, n=40) (Table 1). Smoking remains one of the major risk factors for the development of peripheral vascular disease and the future failure of any revascularization surgery. In this cohort of patients, 49.4% (n=43) were smokers (Table 1) [9]. While the ipsilateral long saphenous vein is the preferred conduit for bypass surgery at our institution, a suitable LSV was unavailable in most patients (75%, n=65) receiving a precuffed graft. In the remaining cases, the GSV was harvested during a prior procedure.

Of the total patients in the study, 26% (n=23) had undergone prior bypass surgery on the same limb, 48% (n=42) required emergency surgery, and among the 85% (n=74) presenting with chronic limb-threatening ischemia, 62% (n=54) exhibited tissue loss (ulceration or gangrene) (Table 1). The popliteal artery served as the distal target for the bypass in 65.5% (n=57) of cases, with the infragenicular popliteal artery utilized in the majority of these instances. Crural arteries were the target in the remaining 34.5% (n=30) of cases (Table 1).

Nearly half of the patients (49.4%, n=43) have a clinical frailty score of 5 and above, considering their comorbidities and surgical history, which indicate that a substantial portion of the patient population was frail, had multiple comorbidities, underwent emergency intervention, and had experienced prior bypass failure (Table 1) [10].

Primary outcome

Amputation-free survival (AFS) was 88.5% (n=77) at one month, 58.6% (n=51) at 12 months, and 47.1% (n=41) at 24 months.

Univariate Analysis

The factors which were included in the univariate analysis are diabetes, hypertension, smoking status, redo surgery, emergency procedure, presence of tissue loss, sex, cardiac disease, target artery, clinical frailty score, and age.

None of those factors showed a statistically significant relationship with the primary outcome, although female patients have a higher amputation rate (hazard ratio {HR} 0.477, CI {0.226-1.005}, p=0.052); however, that didn’t reach a statistically significant p-value. Also, frail patients (patients with a clinical frailty score of >5) showed worse outcomes than others (HR 2.023, CI {0.919-4.453}, p=0.08) (Table 2).

**Table 2:** Univariate and multivariate analyses of amputation using Cox proportional hazards regression are presented. HR: hazard ratio; CFS: clinical frailty score P-value <0.05 is considered significant.

Factor	Univariate HR (CI)	p-Value	Multivariate HR (CI)	p-Value
Diabetic	0.959 (0.452-2.030)	0.913	1.325 (0.490-3.580)	0.579
Hypertension	1.108 (0.512-2.401)	0.794	0.924 (0.361-2.369)	0.870
Smoking status	1.1683 (0.788-3.597)	0.179	4.103 (1.417-11.881)	0.009
Redo surgery	1.286 (0.566-2.922)	0.548	1.436 (0.456-4.519)	0.536
Emergency	1.564 (0.739-3.310)	0.243	1.132 (0.430-2.981)	0.802
Tissue loss	0.519 (0.247-1.090)	0.083	0.217 (0.086-0.55)	0.001
Sex (M vs. F)	0.477 (0.226-1.005)	0.052	0.304 (0.119-0.774)	0.013
Cardiac disease	1.249 (0.593-2.631)	0.559	1.629 (0.562-4.721)	0.368
Target artery (popliteal vs. tibial)	0.850 (0.384-1.881)	0.689	0.890 (0.286-2.773)	0.841
CFS 1-5 vs. 6-8	2.023 (0.919-4.453)	0.080	1.795 (0.286-14.672)	0.585
Age (years)	1.002 (0.959-1.048)	0.920	1.034 (0.963-1.110)	0.360

Multivariate Analysis

The factors that were included in the multivariable analysis are the same ones used in univariate analysis. Smoking status (HR 4.103, CI 1.417-11.881, p=0.009), female sex (HR 0.304, CI 0.119-0.774, p=0.013), and presence of tissue loss (HR 0.217, CI 0.086-0.55, p=0.001) were independent statistically significant predictors of poor outcome of precuffed grafts (Table 2).

Secondary outcomes

Mortality rates were 4.6% (n=3) at one month, 16.1% (n=14) at 12 months, and 20.7% (n=18) at 24 months.

Univariate Analysis

The factors which were included in the univariate analysis are diabetes, hypertension, smoking status, redo surgery, emergency procedure, presence of tissue loss, sex, cardiac disease, target artery, clinical frailty score, and age. Redo surgery demonstrated a statistically significant relationship with survival (HR 2.609, CI 1.097-6.202, p=0.03). Additionally, a history of cardiac disease was statistically significant (HR 3.262, CI 1.250-8.514, p=0.016) (Table 3).

**Table 3:** Univariate and multivariate analysis for survival using Cox proportional hazard regression. P-value <0.05 is considered significant. HR: hazard ratio; CFS: clinical frailty score

Factor	Univariate HR (CI)	p-Value	Multivariate HR (CI)	p-Value
Diabetic	1.027 (0.425-2.482)	0.953	0.269 (0.056-1.288)	0.100
Hypertension	0.699 (0.355-2.002)	0.843	0.651 (0.195-2.172)	0.485
Smoking status	1.050 (0.436-2.528)	0.913	0.748 (0.230-2.432)	0.629
Redo surgery	2.609 (1.097-6.202)	0.030	3.337 (0.742-14.999)	0.116
Emergency	0.748 (0.306-1.833)	0.526	0.578 (0.157-2.2128)	0.410
Tissue loss	1.275 (0.490-3.321)	0.619	4.799 (0.713-32.294)	0.107
Sex (M vs. F)	0.536 (0.225-1.278)	0.159	0.683 (0.208-2.240)	0.529
Cardiac disease	3.262 (1.250-8.514)	0.016	4.708 (1.123-19.729)	0.034
Target artery (popliteal vs. tibial)	0.712 (0.276-1.838)	0.482	2.391 (0.427-13.390)	0.321
CFS 1-5 vs. 6-8	23.309 (0.023-23755.934)	0.373	2.824 (0.640-13.420)	0.987
Age (years)	0.997 (0.947-1.049)	0.899	0.917 (0.819-1.026)	0.131

Multivariable Analysis

The factors that were included in the multivariable analysis are the same ones used in univariate analysis. Only cardiac disease (HR 4.708, CI 1.123-19.729, p=0.034) showed a statistically significant predictor of poor survival outcome (Table 3).

Patency rate

There is no strong evidence to support graft surveillance scan following bypass surgery using synthetic grafts [8]. However, by the end of the second year, 92% had imaging to check whether the graft was still patent. The results showed that 41.4% (n=36) had patent grafts, 50.6% (n=44) had occluded grafts, and 8% (n=7) were missing.

While none of the studied factors reached significant p-value on univariate analysis, the only factors which were found to be statistically significant predictor of poor patency on multivariate analysis were smoking status (HR 2.986, CI 1.285-6.935, p=0.011), presence of tissue loss, (HR 0.318, CI 0.149-0.682, p=0.003), and female sex (HR 0.394, CI 0.182-0.851, p=0.018) (Table 4).

**Table 4:** Univariate and multivariate analyses for graft patency using Cox proportional hazard regression are presented. P-value <0.05 is considered significant. HR: hazard ratio; CFS: clinical frailty score

Factor	Univariate HR (CI)	p-Value	Multivariate HR (CI)	p-Value
Diabetic	1.094 (0.600-1.994)	0.769	1.813 (0.807-4.075)	0.150
Hypertension	1.145 (0.607-2.160)	0.676	0.865 (0.371-2.015)	0.737
Smoking status	1.745 (0.954-3.191)	0.071	2.986 (1.285-6.935)	0.011
Redo surgery	1.079 (0.532-2.186)	0.834	1.117 (0.427-2.925)	0.821
Emergency	1.572 (0.861-2.870)	0.141	1.575 (0.686-3.617)	0.284
Tissue loss	0.631 (0.349-1.141)	0.127	0.318 (0.149-0.682)	0.003
Sex (M vs. F)	0.610 (0.334-1.114)	0.108	0.394 (0.182-0.851)	0.018
Cardiac disease	1.348 (0.742-2.447)	0.327	1.617 (0.665-3.928)	0.289
Target artery (popliteal vs. tibial)	1.153 (0.628-2.118)	0.646	1.141 (0.442-2.945)	0.786
CFS 1-5 vs. 6-8	1.681 (0.403-7.012)	0.476	1.396 (0.303-6.442)	0.669
Age (years)	1.004 (0.970-1.039)	0.827	1.034 (0.977-1.094)	0.250

Postoperative complications

In our study, we identified three major postoperative complications that required surgical intervention. These complications were sepsis secondary to graft infection, bleeding, and pseudoaneurysm formation. The rates of those events were as follows: infection 9.3% (n=8), bleeding 2.3% (n=2), and 4.7% (n=4) had pseudoaneurysm formation.

We conducted a univariate analysis to examine the relationship between graft infection and factors such as diabetes, hypertension, smoking status, redo surgery, emergency procedure, presence of tissue loss, sex, cardiac disease, target artery, clinical frailty score, and age. None of these factors demonstrated a statistically significant prediction of graft infection.

A multivariate analysis was also conducted to identify the risk factors for developing graft infection. However, no statistically significant relationship was found. Nonetheless, diabetic and frail patients (those with a frailty score greater than 5) were at an increased risk of developing infection, with HR 11.214 (CI: 0.748-168.076, p=0.080) and HR 7.803 (CI: 0.692-87.959, p=0.096), respectively (Table 5). A further analysis to determine if any of those complications affect the primary outcome (AFS) was conducted but there is no statistically significant relation.

**Table 5:** Univariate and multivariate analyses for infection using Cox proportional hazard regression. P-value <0.05 is considered significant. HR: hazard ratio; CFS: clinical frailty score

Factor	Univariate HR (CI)	p-Value	Multivariate HR (CI)	p-Value
Diabetic	2.268 (0.540-9.530)	0.264	11.214 (0.748-168.076)	0.080
Hypertension	0.371 (0.089-1.551)	0.174	0.146 (0.014-1.501)	0.106
Smoking status	1.093 (0.272-4.383)	0.901	4.014 (0.278-58.053)	0.308
Redo surgery	1.093 (0.220-5.426)	0.913	0.743 (0.081-6.837)	0.793
Emergency	3.619 (0.730-17.951)	0.115	5.760 (0.358-92.674)	0.217
Tissue loss	0.517 (0.129-2.068)	0.351	0.629 (0.080-4.925)	0.659
Sex (M vs. F)	0.383 (0.095-1.539)	0.176	0.207 (0.020-2.113)	0.184
Cardiac disease	0.911 (0.217-3.826)	0.898	1.682 (0.133-21.282)	0.688
Target artery (popliteal vs. tibial)	1.686 (0.421-6.752)	0.461	1.891 (0.112-31.873)	0.658
CFS 1-5 vs. 6-8	3.124 (0.692-14.096)	0.138	7.803 (0.692-87.959)	0.096
Age (years)	0.998 (0.921-1.082)	0.969	1.058 (0.911-1.229)	0.457

## Discussion

Autologous long saphenous vein remains the best option for lower limb bypasses; however, it is not always available due to previous use or inappropriate size. Other limitations are the associated morbidity from harvesting the long saphenous vein from the contralateral limb or using arm veins. Additionally, the prolonged operative time in patients with underlying comorbidities can be a significant limitation when considering bypass conduits.

Amputation-free survival (AFS) is a key outcome measure when choosing treatment strategies for patients with peripheral arterial disease. In our study, we used AFS as the primary outcome measure. The results after two years of follow-up showed that 47.1% of patients achieved this endpoint, which is comparable to other studies. For example, the study by van der Slegt et al. reported 41% and 43% five-year limb salvage rates when precuffed grafts were used for infragenicular popliteal and crural arteries. Similarly, the study by Loh et al. reported a 55.7% limb salvage rate at three years [4,11].

The result of AFS in our study group was also comparable to vein bypass results, in Best Endovascular vs. Best Surgical Therapy in Patients with Critical Limb Ischemia (BEST-CLI) trail, 42.6% had either major adverse limb event or death from any cause, also 57% reached the amputation free survival end point by the end of three years follow-up in the surgical group of BASIL 1 trial [12,13]. It is worth mentioning that the long saphenous vein was used in 75% of the patients who received surgery in BASIL-1 trial. Although our study design, the heterogeneity of patients, indications, and bypass targets differ from other studies; however, these results indicate that precuffed graft can be used as a valid alternative option when a vein graft is not available.

The adverse effects of smoking and the presence of tissue loss in patient outcomes were obvious. These findings are not limited to precuffed grafts; in one meta-analysis, it was estimated that smoking increases the risks of graft failure two to three times [9]. The presence of tissue loss as an indicator for poor outcome was also noted in other studies, like the study by Taylor et al., which showed that the presence of gangrene is one of the independent predictors of graft failure [14].

Female patients have worse outcomes compared to male patients. This was evident in multivariate analysis, which showed that female sex was an independent predictor of worse primary outcome and patency rate. This was observed in other studies. Kim et al. showed that female sex is associated with a statistically significant increased risk of major amputation following bypass surgery [15].

Although critical limb ischemia is associated with increased overall mortality rate, as observed in the Bypass vs. Angioplasty in Severe Ischemia of the Leg (BASIL) trial, which showed 37% at the end of the study follow-up period [13]. In our study, the presence of cardiac disease, including previous ischemic heart disease, heart failure, and angina, was associated with increased mortality.

In our cohort of patients, nearly half had a clinical frailty score of 5 or more; however, this was not associated with worse outcomes. This finding can encourage the use of the precuffed grafts in frail patients who can’t tolerate a prolonged procedure or morbidity associated with vein harvest. However, a prospective randomized trial will be required to provide strong evidence to support this.

Despite using tibial arteries as bypass target in more than one third of cases, it did not affect the outcome, this may be attributed to increased surgeon experience and the availability of mini-cuff technology, which minimizes the required arteriotomy size and enhance the handling of the graft which might contribute to reduce intimal injury during anastomosis.

There were a few postoperative complications that required surgical intervention, although all of them are known to be associated with prosthetic grafts' uses. In our study, a high infection rate (9.3%) was noted; however, this finding was not associated with adverse effects in amputation-free survival or increased mortality. The surgical intervention consisted of explantation of the old graft with or without reconstruction. The conduits that were used in reconstruction were either biological grafts or upper limb veins. There was no statistically significant relation between postoperative infection and any of the factors that were studied, but diabetic patients and frail patients (clinical frailty score >5) were more likely to develop infection. However, the small sample size limits the ability to draw a relation between the known risks and postoperative complications.

This study has some limitations, it is a retrospective study, lacks a comparative group, and has a small number of patients involved. However, we hope that this study will encourage researchers to conduct a well-designed randomized controlled trial comparing the precuffed grafts with standard vein bypass or prosthetic grafts with a vein cuff.

## Conclusions

This study showed that bypass surgery using a precuffed graft is a valid option in the absence of an autologous conduit, with acceptable results. While the potential benefits of precuffed grafts are intriguing, it is essential that their efficacy be thoroughly evaluated through clinical trials before they can be widely adopted. All proven measures to reduce the risk of infection should be utilized, especially in diabetic and frail patients.
